# Legal Implication in Utilizing Automated Robots: A Written Informed Consent Form Proposal

**DOI:** 10.1002/rcs.70064

**Published:** 2025-04-22

**Authors:** Maria Teresa Contaldo, Sonia Triggiani, Giacomo Vignati, Daniele Bracchi, Gianpaolo Carrafiello

**Affiliations:** ^1^ Postgraduation School in Radiodiagnostics University of Milan Milan Italy; ^2^ Agnoli e Giuggioli Law Firm Milan Italy; ^3^ University of Milan International Law Milan Italy; ^4^ Radiology and Inverventional Radiology Department Fondazione IRCCS Cà Granda Policlinico di Milano Ospedale Maggiore Milan Italy

**Keywords:** clinical practice, doctor‐patient relationship, informed consent, interventional radiology, liability, robotic systems

## Abstract

**Background:**

Robotic systems enhance physicians' capabilities by replicating hand movements in real‐time, ensuring precise control and a quick return to conventional procedures if patient safety is compromised. Physicians performing robot‐assisted procedures bear ultimate responsibility, sharing potential liability with manufacturers for malfunctions.

**Methods:**

This study, conducted by a transdisciplinary team of interventional radiologists and a legal expert, evaluates the integration of robotic systems in interventional radiology through a comprehensive literature review, addressing potential legal contingencies.

**Results:**

This paper aims to define liability in this context and examines how workflows and doctor‐patient relationships might be reshaped: patients must be informed about treatment options, including details about robot‐assisted procedures and associated risks.

**Conclusions:**

These systems could significantly impact interventional radiology practice. A dedicated informed consent process is necessary to ensure clear communication and protect the decision‐making process and patient‐centred care; thereby, an informed consent is proposed to comprehensively address these needs.

## 1 Introduction

Robotic systems, equipped with advanced decision‐making capabilities, data analytics, and automation, can offer invaluable support to healthcare providers in diagnosing, treating, and managing patient conditions. However, as these systems become increasingly involved in clinical decision‐making, questions arise about how they might impact the core principles of medical care.

The rapid progression of robotic technology has considerably facilitated its integration into the realm of Interventional Radiology (IR). While robotics has already gained significant prevalence in surgical fields, its adoption in IR has outpaced the existing legal frameworks established for more conventional medical practices. These legal structures, which struggle to keep pace with the rapid advancement of technological innovations, now face the challenge of keeping up with the rapid integration and distinctive applications of robotics within the context of IR. This gap in regulatory frameworks is significant, as the increasing use of robotics in medicine introduces complex issues related to liability, patient privacy and decision‐making.

It is essential to underline that legal frameworks must align with technological innovations, ensuring that the integration of robotics into healthcare is done in a manner that is safe and beneficial to patient care. It is also important to understand that although Artificial Intelligence (AI) has the capacity to analyse large datasets and might reveal insights more quickly and deeply than humans, it lacks the empathy, judgement, and comprehensive understanding that a clinician has in interacting with patients. AI's capabilities are purely analytical and do not encompass the human qualities essential to healthcare [1]. Therefore, integrating AI tools should be about augmenting the clinician's capabilities and not replacing them. By doing so, healthcare can benefit from the strengths of both AI and human judgement, leading to improved outcomes, innovation, and a trust‐based relationship between patients and healthcare providers.

The aim of this study was to analyse the legal implications that may arise when a complication occurs during a remote interventional procedure performed by an operator with the robot on‐site.

If an automated robot makes an error during a procedure or malfunctions, determining liability becomes a complex issue. It may involve the manufacturer, the healthcare institution, and the healthcare professionals involved.

## 2 Methods

We established a transdisciplinary consortium composed of experienced medical professionals, including an interventional radiologist and resident physicians under their supervision, alongside a legal expert specialising in healthcare and international law. This team was carefully assembled to address the complex challenges associated with integrating robotic systems into medical procedures.

The primary objective of this consortium is to design and implement an informed consent process that comprehensively safeguards the interests of both operators and patients. The proposed informed consent is meticulously structured to account for various contingencies that may arise during robotic procedures. It explicitly outlines protocols to be followed in the event that a remote procedure must be transitioned to an on‐site intervention. Such transitions may be necessitated by unforeseen technical malfunctions of the robotic equipment or by the emergence of complications related to the robotic procedure itself.

By addressing these potential issues in advance, the informed consent aims to ensure clarity and readiness, thereby enhancing patient safety and operator accountability. This proactive approach not only protects patients' rights and ensures their informed participation but also provides a legal shield for medical professionals, thereby fostering a secure medical environment. This informed consent proposal did not require ethical approval.

## 3 Discussion

The field of IR is distinguished by the continual implementation of cutting‐edge technological minimally invasive innovations.

Every interventional procedure, either endovascular or percutaneous, can be performed by robotic systems. Consequently, the integration of robotics with IR practice signifies an exciting evolution stemming from substantial advancements in medical technology and engineering in recent years. This incorporation of robotics enhances precision, improves procedural safety, and offers greater control during complex interventions. Furthermore, robotic systems in IR pave the way for innovative treatment approaches, potentially reducing recovery times and minimising patients' discomfort. These advancements not only reflect a leap forward in interventional capabilities but also underscore a collaborative triumph between medical science and engineering, heralding a new era in patient care and procedural efficiency [2].

Given that, robotics manifest as the physical implementation of AI and the automated robots (ARs) are appropriately categorised as medical devices: these robotic systems, which physically interact with patients and assist in surgical procedures, bridge the gap between computational AI algorithms and real‐world medical applications. As such, they transcend being mere technological innovations to become integral tools in patient care, also in IR, and therefore become subject to the stringent quality and safety standards that govern all medical devices.

Staying aligned with regulations guarantees that the benefits of robotic assistance in IR, such as enhanced precision in patients' outcomes, are realized without compromising safety and reliability.

### 3.1 Liability and Accountability Issues

This designation of ARs as medical device also highlights the need for continual oversight and maintenance, ensuring that they operate effectively and evolve alongside advancements in both AI and medical technology. Regular updates and maintenance in accordance with the latest standards are also crucial in this dynamic field of medical technology. In the United States, this means adhering to the Food and Drug Administration (FDA) guidelines, whereas in Europe, it involves following the European Conformity (CE) standards. Both manufacturers and users of these robotic systems must rigorously follow these regulations to ensure the robots' safety and effectiveness.

Manufacturers are responsible for designing and producing robots that meet these strict regulatory requirements. Meanwhile, medical professionals using these systems must also comply, ensuring safe and proper usage.

The employment of ARs in IR introduces a spectrum of legal issues that demand thorough examination and understanding. As technology advances, legal frameworks may need to adapt to encompass the unique challenges posed by robotic interventions: the incorporation of advanced technology in medical procedures necessitates a careful analysis of liability, which may vary significantly across different jurisdictions.

Questions regarding responsibility for malfunctions or errors, whether attributed to the machine's programming or operational use, become paramount, especially in cases where robotic assistance is integral to patient care. Privacy concerns, especially with data handling and patient confidentiality in the context of AI and robotics, also emerge as critical legal considerations. Furthermore, informed consent processes must evolve to adequately inform patients about the role and implications of robotic assistance in their treatment. Patients should be made aware of the benefits, risks, and any differences in procedure when robots are involved compared with traditional methods.

At present, there is a noticeable absence of well‐defined regulations specifically tailored to address the legal challenges arising from the incorporation of ARs in healthcare settings. The development of new guidelines and policies is mandatory in order to effectively govern these new robotic‐assisted scenarios. Issues such as determining accountability in the event of a robotic malfunction or error, safeguarding patient data handled by these systems, and ensuring standards in robotic programming and operation are all critical areas that require regulatory attention.

Clear contractual agreements and liability clauses should be established between the robot manufacturer, healthcare providers, and other relevant parties. Moreover, there is a need for international collaboration to establish universal standards and protocols for the use of robots in healthcare. A global approach would not only facilitate consistency in legal practices but also help in sharing knowledge and experience across borders, enhancing the safe and effective implementation.

Various nations, including EU member states, have introduced new regulations and adjusted overarching liability frameworks for AI. These measures aim to enhance clarity, transparency, and public confidence in automated solutions within the healthcare setting [3].

The European Resolution dated February 16th, 2017 could serve as the initial framework for regulating robotics: a potential approach involves conferring legal personhood upon robots, thereby holding them liable for any resultant harm and enabling them to compensate victims [4].

Culpability is tied to punishment, and a computer or robot cannot be subject to penalty, as it lacks an understanding of civil liberties [4]. Thus, identifying the individuals accountable becomes imperative.

According to O′ Sullivan et al. [4], as for the existing legal regulations derived from challenges encountered in the automotive sector, a robot is not legally liable for its actions.

In the case of telae‐operated robots, the IR is an obvious candidate, unless the cause of death is attributed to a machine malfunction. Signal loss during telae‐procedure prompts considerable deliberation on whether autonomous procedure should be activated under such circumstances.

It may be necessary for regulatory authorities to adjust their guidelines to incorporate the utilization of ARs in medical procedures.

Regulatory bodies also need to update or establish guidelines for the training and certification of professionals using such technology: healthcare professionals in IR (radiologists, nurses, radiographers) using these ARs must be appropriately licenced and credentialled [5].

The establishment of industry standards for training programs and certifications is vital to guarantee the safe and efficient utilization of these technologies.

It is essential to recognise that the legal framework for ARs in healthcare is continuously developing. Healthcare providers, manufacturers and regulators need to collaborate effectively to address the emerging challenges and safeguard patients' safety in this evolving field [5, 6].

Manufacturers need to be aware of the evolving standards and regulations that impact the design and deployment of these robots. Similarly, healthcare providers must understand their responsibilities and liabilities in using these advanced technologies in patient care.

Engaging legal professionals who specialise in healthcare law is highly recommended to navigate this complex and changing legal territory. Such experts can offer comprehensive guidance, ensuring that all parties involved are informed about current legal requirements and best practices. They can also provide valuable insights into potential future legal trends and how to prepare for them.

This approach ensures that the integration of ARs in healthcare is conducted responsibly and in line with the latest legal standards, prioritising patient safety.

Moreover, extending this collaboration to include patient's advocacy groups and regulatory committees can further enrich the dialogue, ensuring that patients' rights are at the forefront of any decisions. This inclusive approach can lead to more robust and patient‐centric policies, enhancing the safe implementation of ARs in healthcare.

In consideration of the aforementioned points, the demand for ‘*explainable AI*’ in robotics is becoming increasingly critical. The application of this topic extends beyond technical aspects to include educational and routine operational uses. The presence of explainable robotics in the medical field would significantly enhance trust among healthcare professionals as it provides clarity on how robotic decisions and actions are made [4].

### 3.2 Doctor‐Patient Relationship Paradigm

While AI‐based decision‐making tools might demonstrate advanced knowledge and analytical capabilities, it is widely considered crucial to maintain the clinician's role as the paramount decision‐maker in the healthcare process. This concept, known as maintaining the ‘*human‐in‐the‐loop*’, ensures that the professional integrity and judgement of the clinician guide the final decisions [3]. Upholding this principle is not only a matter of preserving the human element in care but also ensuring accountability, transparency, and personalised patient care. Having a *human‐in‐the‐loop* approach provides an additional layer of oversight, which is especially important in complex or ambiguous cases where AI algorithms might not have all the necessary context. It also allows for the integration of patient preferences and values in decision‐making, something that AI tools might not adequately consider.

ARs have the capability to gather and process highly sensitive patient data, which necessitates rigorous adherence to stringent data privacy guidelines [5]. These guidelines, exemplified by the Health Insurance Portability and Accountability Act (HIPAA) [7] in the United States and the General Data Protection Regulation (GDPR) [8, 9] in Europe, play a paramount role in safeguarding patient information from any form of unauthorized access, unauthorized disclosure, or breaches of confidentiality. Complying with these guidelines is not only a legal requirement but also an effort to maintain the utmost integrity and security of patient data throughout its lifecycle within the healthcare ecosystem.

Inquiries regarding patient autonomy, the necessary details for informed consent customised to robotic procedures, and the potential consequences for the patient‐physician relationship hold considerable significance. The integration of decision‐making tools based on robotic systems has the potential to reshape the traditional two‐way interaction between patients and physicians, giving rise to a more complex three‐way engagement.

The primary critical concern is centred on the substantial influence that robotic systems are projected to exert on the fiduciary relationship existing between physicians and patients, deeply rooted in trust, transparency, and responsibilities of healthcare professionals.

The fiduciary relationship between physicians and patients is characterised by a duty of care and an obligation to act in the best interests of the patient. Physicians are entrusted with making informed decisions, guided by their medical expertise and a deep understanding of the patient's individual needs and preferences. The introduction of robotic systems into this equation introduces new complexities. The utilization of *black‐box algorithms* has the potential to erode the established level of trust inherent in the patient‐physician relationship. Consequently, the emerging trilateral relationship should not exclude essential humane relational practices in any manner.

Healthcare professionals must navigate the dual responsibility of integrating technology while preserving the essential elements of personalised care and shared decision‐making. Physicians are required to establish their positions as intermediaries positioned between patients and systems, with a dedicated commitment to preserving the patient's viewpoint, values, and apprehensions as the pivotal elements guiding the decision‐making procedure.

Hence, users of ARs have the responsibility to employ their judgement and empathy to take into account social, personal, and clinical contexts beyond the capabilities of the robots they utilise in order to obtain *patient‐tailored* results.

### 3.3 Minimally Invasive & Automated Robotic‐Assisted Procedures Informed Consent

Minimally invasive procedures, while providing several advantages such as reduced trauma and expedited recovery, inherently carry a certain level of risk, including the potential requirement for transitioning to a more invasive procedure. The decision to shift to a more extensive approach, in a surgical setting, is contingent upon a multitude of factors, including the patient's anatomical characteristics, the surgeon's judgement and the specific clinical circumstances encountered during the course of the procedure.

These principles and considerations regarding minimally invasive procedures are universally applicable to automated robotic‐assisted procedures: in situations marked by complexity or in the early stage of adoption of innovative techniques, a certain degree of conversion to well‐established treatments may occur and it is universally deemed acceptable.

Therefore, prior to the initiation of any procedure, healthcare practitioners have a fundamental responsibility to engage in comprehensive discussions with patients, encompassing treatment alternatives and potential outcomes. This communicative exchange represents an integral component of their professional relationship, formally underpinned by a well‐documented informed consent procedure designed to ensure that patients possess a comprehensive understanding of the proposed medical intervention along with its associated risks and benefits [8, 9]. If this level of detail is lacking, the physician could face challenges in evaluating the comprehensiveness of the patient's consent for the recommended treatment, potentially leading to a deficiency in clarity and mutual understanding. Therefore, acknowledging and addressing the potential for conversion during the informative consultation process is of paramount importance; this ensures that the patient is well‐informed about the full range of possibilities and potential contingencies associated with the chosen treatment plan.

In complex cases, where the pathology or the patient's condition presents unique challenges, interventional radiologists may encounter the need to adapt their approach during the procedure, potentially necessitating a conversion to a more conventional technique for the sake of patient safety and optimal outcomes. Similarly, as the ARs user gains experience with these innovative techniques, there may be a period of adjustment where conversions could occur as part of the learning process.

Acknowledging and managing these situations with flexibility and expertise is integral to maintaining patients' safety and achieving successful outcomes. Therefore, while a certain level of intraoperative conversion rate is acceptable, efforts should continually be made to minimise such occurrences through ongoing training, careful patient selection, and refined surgical techniques.

Such a comprehensive approach promotes transparency and trust in the patient‐physician relationship, ultimately contributing to more patient‐centred and well‐informed healthcare decision‐making. Additionally, this empowers patients to actively participate in their healthcare journey and fosters a sense of trust and collaboration between patients and healthcare providers, ultimately contributing to improved outcomes and overall satisfaction with the healthcare experience.

Effective communication has been established as a pivotal factor in influencing patients' decisions to pursue legal actions in response to medical errors [10]. The quality of communication between healthcare providers and patients can significantly impact the patients' perception of the care they received and the subsequent actions they may take. Patients who feel that their concerns were heard, that they were provided with clear and honest information about the error, and that they received empathetic and compassionate support from their healthcare providers are less likely to consider legal recourse.

It is imperative to respect each patient's autonomy, allowing them to make decisions aligned with their preferences and values. Specific patients may elect ‘watchful waiting’ as their preferred approach instead of pursuing aggressive open surgery, citing potential advantages such as reduced scar size and minimised post‐operative discomfort. Conversely, there are those who deliberately opt for invasive but well‐established procedures. It is essential to underscore that irrespective of their choices, all patients should have access to comprehensive information and appropriate preparation [11, 12, 13].

Nonetheless, the inherent challenges lie in the establishment of the insufficiency of the treatment, the potential infringement upon the patient's rights or the intricate assessment of legal causality within the harm inflicted by the algorithms and their subsequent chain of events [3]. This task demands a meticulous examination and rigorous validation of multifaceted factors contributing to potential treatment inadequacies, coupled with a thorough scrutiny of the causality stemming from algorithms and the interplay between clinical decisions, patients' rights, and their subsequent consequences.

### 3.4 Written Informed Consent Form Proposal

It is advisable to articulate and explicitly detail patients' preferences regarding treatment alternatives and their corresponding potential consequences, such as the possibility of conversion to a more invasive procedure, instead of relying solely on a generic all‐encompassing consent for treatment involving automated robot assistance [10]. A comprehensive consent process should ensure that patients receive specific and tailored information about the nature of the treatment, the role of automation and robotics, potential benefits, anticipated outcomes, as well as any associated risks or complications. It is deemed unacceptable for patients to be confronted with an entirely unforeseen and unanticipated situation, if this occurrence can be prevented through a more thorough and comprehensive informed consent procedure.

In every circumstance, regardless of how remote the likelihood of an event of conversion may be, it is inadequate to warrant the omission of informing patients about the potential for such an event and the absence of seeking patients' explicit consent (or refusal) regarding the potential procedural alteration. This fundamental principle underscores the importance of transparent and candid communication in healthcare, as previously emphasised.

Interventional radiologists, given their specialised knowledge and experience, should maintain the autonomy to exercise their professional judgement when assessing the necessity for transitioning to an alternative technique in response to changing clinical circumstances. Furthermore, there should exist an option to abstain from performing a procedure when patients have clearly communicated their preference to prevent the conversion of their robot‐assisted procedure to a different approach.


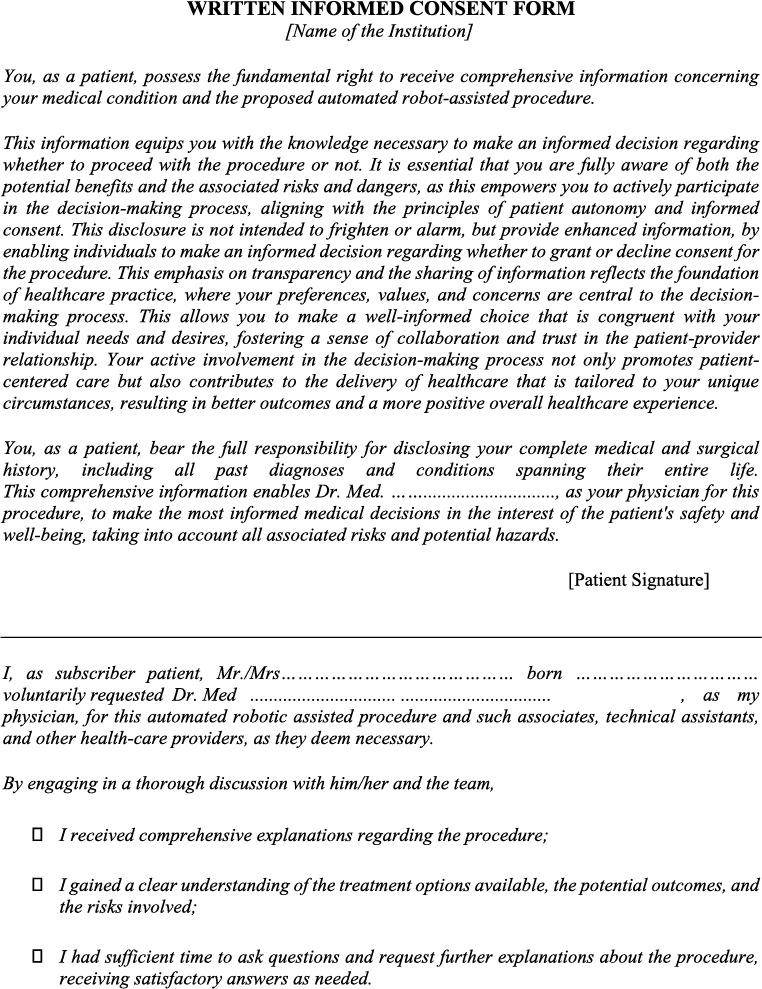



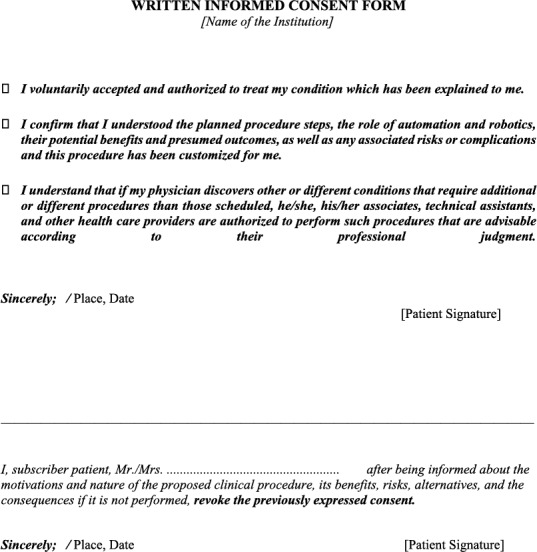


## 4 Conclusion

In utilising the sophisticated analytical power of AI‐based tools, it is imperative to preserve the collaborative process between patient and physician. Patients should maintain their autonomy by making informed decisions with the guidance of physicians. This ensures a shared decision‐making process that respects patient values and preferences while enhancing diagnostic accuracy and therapeutic precision. This equilibrium is crucial as it allows patient care to derive benefits from technological advancements while also valuing the unique and irreplaceable aspects of human clinical judgement and a patient‐focused approach in healthcare.

At the same time, healthcare providers and institutions must prioritise clear, empathetic and transparent communication. By implementing a comprehensive and personalised informed consent procedure, they can equip patients with a more profound comprehension of their treatment choices and the potential consequences associated with the use of automated robotic assistance.

This not only respects patients' autonomy but also aligns with the principles of patient‐centred care, fostering transparency and trust in the doctor‐patient relationship.

Future developments are centred on the creation of strong systems and protocols to consistently ensure patients' privacy and data security in the implementation and use of ARs technology in healthcare. Expanding upon this perspective, the implications of robot‐human interactions in clinical settings should also be carefully examined.

## Author Contributions

All authors contributed significantly to the planning and design of the study, the drafting of the manuscript, and the critical revision of important intellectual content. Each author has approved the final version of the manuscript and agrees to be accountable for all aspects of the work.

## Ethics Statement

The authors have nothing to report.

## Conflicts of Interest

The authors declare no conflicts of interest.

## Data Availability

Data sharing not applicable to this article as no datasets were generated or analysed during the current study.
